# Rhinophototherapy, an alternative treatment of allergic rhinitis: Systematic review and meta-analysis

**DOI:** 10.1016/j.bjorl.2020.12.016

**Published:** 2021-02-16

**Authors:** Tatyana Machado Ramos Costa, Fernanda Melo Carneiro, Karen Amanda Soares de Oliveira, Maria Fernanda Barbosa Souza, Melissa Ameloti Gomes Avelino, Isabela Jubé Wastowski

**Affiliations:** a Universidade Estadual de Goiás (UEG), Campus Anápolis de Ciências Exatas e Tecnológicas Henrique Santillo, Anápolis, GO, Brazil; b Universidade Estadual de Goiás (UEG), Campus Laranjeiras, Goiânia, GO, Brazil; c Universidade Federal de Goiás (UFG), Faculdade de Medicina (FM), Goiânia, GO, Brazil; d Universidade Federal de Goiás (UFG), Hospital das Clínicas (HC), Goiânia, GO, Brazil

**Keywords:** Phototherapy, Intranasal irradiation, Allergic rhinitis, Rhinophototherapy, Hay fever

## Abstract

**Introduction:**

Allergic rhinitis is a chronic inflammatory disease of the nasal mucosa, mediated by immunoglobulin E, affecting 1 in 6 individuals. The treatment aims at attaining symptomatic control with minimal side effects, a requirement for new alternative therapies, including phototherapy, as it has an immunosuppressive and immunomodulatory effect.

**Objective:**

To identify the effectiveness of phototherapy in the treatment of allergic rhinitis through a meta-analysis.

**Methods:**

We searched Web of Science, Scielo, PubMed, SCOPUS, PEDro, and LILACS databases, using the terms: “intranasal irradiation”, “phototherapy” and “allergic rhinitis”. The R software Metafor package was used for the meta-analysis and the effect size was calculated for each symptom individually.

**Results:**

All symptoms decreased considerably after phototherapy: rhinorrhea (ES• = −1.35; *p* < 0.0001; I^2^ = 91.84%), sneezing (ES• = −1.24; *p* <  0.0001; I^2^ = 91.43%), nasal pruritus (ES• = −1.10; *p* < 0.0001; I^2^ = 91.43%); nasal obstruction (ES• = −1.11; *p* < 0.0001; I^2^ = 91.88%). The effects were more significant in perennial allergic rhinitis than in the seasonal type.

**Conclusion:**

Considering the effect size and the statistical significance attained in our study, rhinophototherapy showed to be an effective treatment for reducing the nasal symptom scores triggered by AR.

## Introduction

Allergic rhinitis (AR) is a type of chronic nasal mucosa inflammation mediated by immunoglobulin E (IgE) and induced by allergens, which affects one in six individuals worldwide.^1^, ^2^, ^3^ The etiology of AR is determined by a combination of genetic, environmental and familial predisposition factors. The symptoms are triggered by seasonal or perennial allergens that cause continuous or intermittent complaints of sneezing, rhinorrhea, nasal pruritus, palatal pruritus, nasal congestion and palpebral edema. These symptoms have an impact on sleep, concentration, learning, work and leisure activities, compromising the quality of life, in addition to being one of the risk factors for asthma.^4^, ^5^, ^6^, ^7^

The pathophysiology of the disease is characterized by an initial phase of sensitization to a specific allergen. After the exposure, individuals sensitized to the allergen show specific immune responses. The activation of T helper 2 (Th2) cells plays a role in the disease onset and maintenance.^4^, ^8^, ^9^ Eosinophils, mast cells and basophils, which are innate immune response cells, are considered the main effector cells of AR. These cells release inflammatory mediators, such as histamine, prostaglandins, cytokines, tryptase, leukotrienes and eosinophilic cationic protein. These mediators are responsible for most of the pathological processes that occur in the nasal mucosa.^4^, ^7^, ^10^

The treatment of AR is a symptomatic one. Among the treatment measures, the following stand out: environmental control (avoiding the allergens), drug administration (antihistamines, topical and systemic nasal steroids, anticholinergics and leukotriene antagonists), acupuncture, immunotherapy and phototherapy.^4^, ^11^, ^12^ Although new antihistamines and local steroids have been used with good results, there are cases where complete symptom remission cannot be achieved. Moreover, the use of these drugs is controversial in special patient subgroups, such as pregnant and lactating women. All of these factors justify the need to seek new effective treatment options.^2^, ^11^, ^13^

Rhinophototherapy, due to its immunosuppressive and immunomodulatory effect, is a promising and non-invasive treatment option for several immune-mediated pathologies, also representing a therapeutic option for patients with perennial or seasonal AR.^2^, ^14^, ^15^ Phototherapy is able to inhibit the effector phase of allergic reactions, inhibit histamine release induced by mast cell antigens and induce apoptosis in T lymphocytes and eosinophilic cells, reducing the Cytopathic Effect (CPE) and Interleukin 5 (IL5) production.^7^, ^16^, ^17^

In the 1980s and 1990s, studies were carried out on the effects of monochromatic light (laser and LED) on biological tissues, i.e., biomodulation, which is a photochemical effect of the absorption of light by biological tissue.^18^ Experimental studies carried out at the cellular level have shown that both the laser (Light Amplification by Stimulated Emission of Radiation) and the LED (Light-Emitting Diode), at the same wavelengths, intensity and time of irradiation, have similar biological effects.^18^, ^19^

Thus, although seldom mentioned in the literature, treatments that use phototherapy have been developed in several research centers worldwide to address immune-mediated diseases, including AR. Hence, considering the high prevalence of AR, and the low frequency of citations of this non-drug therapy, we ask: Is phototherapy effective in reducing nasal scores in adults and/or children with allergic rhinitis, in comparison to conventional or placebo treatment?

## Methods

The protocol for conducting the study was registered on the PROSPERO platform under CRD: 42020147542. The review methods were established before the beginning of the research, and during the process, it was established that randomized and non-randomized studies would be included. The inclusion of both types of studies is because the same research questions were addressed in both types of clinical trials, and the limitation of this review to randomized clinical trials (RCT) would provide us with an incomplete summary of important effects related to the assessed treatment.

The search strategies, developed according to each database, and data collection were carried out in the months of June and July of 2019. The PubMed, Scielo, Web of Science, LILACS and the PEDro databases were used in the search. For the assessment regarding the inclusion of articles in the study, 03 (three) evaluators (T.M.R.C., K.A.S.O., M.F.B.S.) independently performed the analysis and in two stages. In the first stage, the title and abstract of the studies were assessed and those containing the terms “phototherapy”, “intranasal irradiation”, “allergic rhinitis” were selected. In the second stage, the studies identified in the first stage were retrieved, read in full and data were extracted from the selected articles. Any discrepancies were resolved by consensus.

The selected articles should have the terms: “phototherapy”, “intranasal irradiation”, “allergic rhinitis”, *fototerapia* and *rinite alérgica* in their title, abstract or keywords. There were no restrictions regarding language and date of publication. The studies should compare the results of the treatments using phototherapy as an intervention, through the evaluation of before and after the therapy or treated group versus a control group (placebo or antihistamine). Studies with more than eight participants, of any age, of both genders who received endonasal phototherapy were included, in addition to studies that evaluated the effects of endonasal phototherapy using any irradiation method. There were no restrictions related to the dose, duration or frequency of sessions.

Studies in which the participants had any significant abnormalities in the nasal structures, asthma, respiratory tract infection in the previous two weeks, or lower respiratory infection in the four weeks before the study were excluded, as well as studies in which participants used medications such as antihistamines and nasal decongestants (1 week before the start of the study), topical corticosteroids or cromolyn sodium (2 weeks before the beginning of the study), corticosteroids (4 weeks before the beginning of the study), immunotherapy in the last 2 years before the study.

Studies whose clinical results of interest were not clearly reported with quantifiable data or when it was not possible to extract and calculate the appropriate data from the published results, were excluded from the research. Standard clinical files and spreadsheets were used to integrate and organize the studies using the Excel software.

The main analyzed result was symptom assessment (rhinorrhea, sneezing, nasal pruritus and nasal obstruction) using a self-reported analog scale (0: no symptoms; 1: mild symptoms; 2: moderate symptoms; 3: severe symptoms) and the Total Nasal Symptom Score (TNSS) scale. The secondary results included quality of life assessment using the Rhinoconjunctivitis Quality of Life Questionnaire (RQLQ), which were analyzed in this review to assess the effects of rhinophototherapy on the quality of life of the individual with AR.^2^, ^4^, ^10^, ^13^^,^^20^

Searches were carried out on the Sucupira platform’s database of theses and dissertations and in the consensuses (Allergic Rhinitis and its Impact on Asthma – ARIA, International Consensus Statement on Allergy and Rhinology: Allergic Rhinitis – ICAR and European Position Paper on Rhinosinusitis and Nasal Polyps – EPOS) regarding the use of phototherapy as AR treatment. However, no evidence was found in the consensuses and due to the difficulty in standardizing the search strategy and the lack of some complete documents in the database, it was decided not to include gray literature.

The risk of bias assessment of the included studies was carried out using the Cochrane Risk of Bias Tool: ROB-2.0 (Risk Of Bias tool for randomized trials) and ROBINS-I (Risk Of Bias tool to assess Non-randomized Studies of Interventions). The studies were scored as mild, medium and high risk of bias in the domains assessed by each tool.

The R software Metafor package was used for the meta-analysis.^21^ First, the effect size (consistency of effects across studies) was calculated for a random effect and was based on the average of the difference in the score generated before and after treatment or for the control group and treated group. Therefore, independent groups and paired groups were added to the same meta-analysis.

To calculate the effect size, the mean, standard deviation and sample size were removed from each study. The random effect was used, since the effect may vary from study to study (e.g., different groups between studies – age, gender). The weight (wi) of each study was calculated, so studies with a higher sample N and lower standard deviation have greater weight and contribute more to the accumulated effect size. Moreover, the variance (Q) between the studies and the heterogeneity (I^2^), in which the proportion of the observed variance reflects a real difference between the effect sizes, were calculated.

The I^2^ statistic was used to infer the percentage of variance attributed to heterogeneity. The ​​ I^2^ values vary between 0% and 100%, with the 0% value indicating absence of heterogeneity, values between 25% and 50% indicating low heterogeneity, values between 50% and 75% indicating moderate heterogeneity and I^2^ > 75% indicating high heterogeneity.^22^, ^23^

The effect size was calculated for each type of symptom separately: rhinorrhea, sneezing, nasal pruritus and nasal obstruction. When the observed heterogeneity (I^2^) was very high, there was an attempt to explain part of this variation through a group meta-analysis or meta-regression. Treatment time in days, initial dosimetry, final dosimetry and the number of weeks of treatment were used as moderators. To explain the observed heterogeneity, subgroup analyses were carried out regarding the type of AR as perennial or seasonal and as to the type of study design as RCT or non-randomized, pairing and blinding in the studies.

To identify whether the data used in the meta-analysis were influenced by publication bias, the funnel plot was plotted for each meta-analysis performed using the funnel command of the Metafor package in the R program.^21^ The funnel plot is a scatter plot of the effect size by the sample size or variance and the asymmetry indicates that the distribution is not homogeneous and that the effect size is most likely being influenced by the presence of bias in publications. Its use is best employed for meta-analyses with more than 30 studies.^22^

To identify the robustness of the meta-analysis and the number of studies needed to modify the significance of the effect size, fail safe N^24^ was calculated using the fsn command in the Metafor package. To verify whether the effect sizes follow a normal distribution, the Q–Q plot was performed using the qqnorm command of the Metafor package, in which the observed quantiles of the effect size distribution are plotted against the quantiles of a normal theoretical distribution. If the observed data has a normal distribution, the points fall close to the line.^21^

## Results

**Figure 1:**
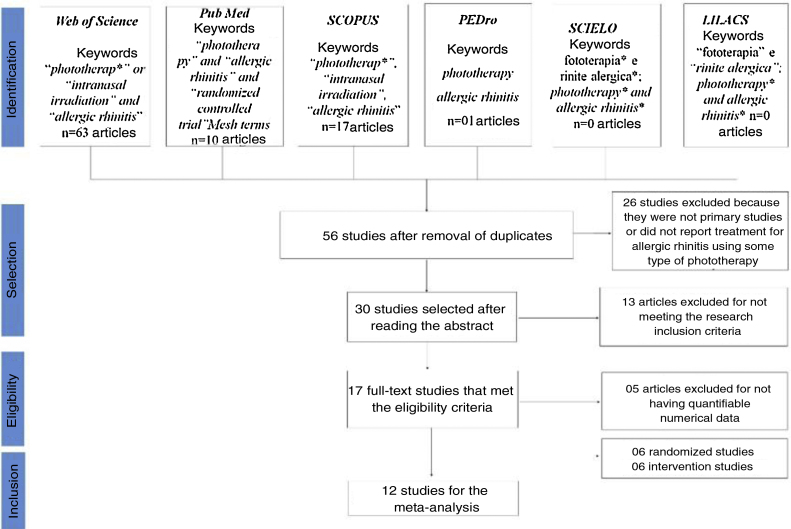
PRISMA flowchart from the identification stage to the inclusion phase of studies that were eligible for the meta-analysis of endonasal phototherapy in the treatment of AR.

Of the 56 articles selected from the databases, 17 met the eligibility criteria for the systematic literature review and 12 articles had the necessary data to perform the statistical evaluation (mean and standard deviation) (Fig. 1).

In these 12 studies used for the meta-analysis of nasal scores before and after phototherapy, the diagnosis for AR was confirmed through the skin prick test and/or specific test for IgE. Studies on perennial AR and seasonal AR with a sample of 323 participants in randomized studies and 126 participants in intervention studies were evaluated.

**Figure 2:**
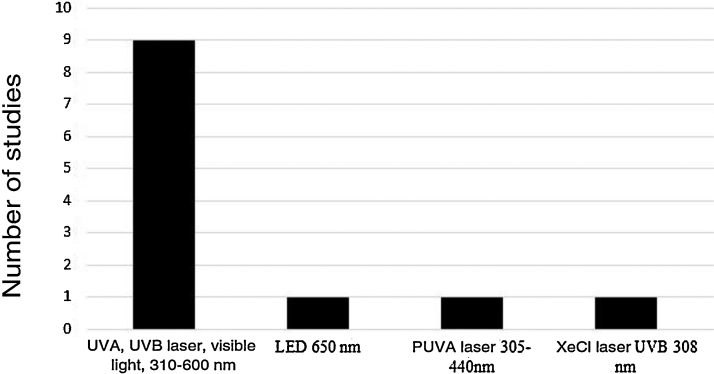
Frequency of phototherapy spectrums used in the studies.

Five device models found on the market were employed (Rhinoligth, Bionase, Rhine Care, 660−850 nm LED, XeCl UVB excimer laser), which emitted five light spectrum bands. A specific protocol was used for each device: dosimetry in joules per application, application time per session, time of treatment per week and number of weeks for treatment. The light spectrums used in the treatments were: UVA laser (25%), UVB (5%), visible light (70%), 310–600 nm; 650 nm LED; 305–440 nm psoralen with ultraviolet light A (PUVA) laser and 308 nm XeCl UVB laser (Fig. 2).

All studies evaluated the effectiveness in reducing nasal symptoms of AR and / or the quality of life score of the sample assessed before and after treatment and/or in relation to the control group, placebo (low-intensity visible light or white light) or antihistamine (Table 1).

**Table 1:** Characteristics of the analyzed studies and risk of bias.

Author/year	N	Type of phototherapy	Study objective	Study conclusion	Study design	Risk of bias
Sematonyte et al., 2018^25^	15	UV-A (25%), UV-B (5%) laser, visible light (70%), 310–600 nm	To investigate the effect of endonasal phototherapy on the quality of life of patients with perennial AR.	Endonasal phototherapy can relieve nasal symptoms and improve quality of life of patients with moderate/severe AR.	Prospective	High
Bella et al., 2017^13^	25	UV-A (25%), UV-B (5%) laser, visible light (70%), 310–600 nm	To evaluate whether endonasal phototherapy is safe and effective in the treatment of perennial AR.	Endonasal phototherapy is an effective and safe treatment in perennial AR.	Prospective, randomized, double-blind, placebo controlled	High
Alyasin et al., 2016^10^	62	UV-A (25%), UV-B (5%) laser, visible light (70%), 310–600 nm	Treat AR with phototherapy in patients who are unresponsive to treatment with antihistamines or in those whose work contraindicates the use of the drug, or who do not use medication.	Endonasal phototherapy was an effective treatment in patients with AR. However, a grounding study is still recommended.	Prospective, randomized and blind	High
Lee et al., 2013^26^	42	LED 650 nm	To evaluate the safety and efficacy of low energy phototherapy in patients with perennial AR.	Phototherapy is effective for the treatment of perennial AR and is a therapeutic option in the treatment management without the use of steroids, of immune-mediated diseases of the nasal mucosa	Open clinical study	High
Albu and Baschir, 2013^20^	77	UV-A (25%), UV-B (5%) laser, visible light (70%), 310–600 nm	To compare the effectiveness of rhinophototherapy and azelastine hydrochloride for the treatment of seasonal AR.	Both rhinophototherapy and azelastine hydrochloride are capable of significantly improving TNSS.	Randomized, prospective	Medium
Yildirim; Apuhan e Kocoglu, 2013^27^	31	UV-A (25%), UV-B (5%) laser, visible light (70%), 310–600 nm	To evaluate the effect of endonasal phototherapy on the microbial flora in a patient with AR.	Endonasal phototherapy does not change the aerobic microbial flora of the nasal mucosa in patients with perennial AR.	Prospective, self-compared and blind.	High
Garaczi et al., 2011^12^		UV-A (25%), UV-B (5%) laser, visible light (70%), 310–600 nm	To compare the efficacy between endonasal phototherapy and fexofenadine hydrochloride, in the treatment of seasonal AR.	Endonasal phototherapy is more effective than fexofenadine hydrochloride in reducing clinical symptoms of seasonal AR.	Randomized prospective,	High
Bremher and Schön, 2011^28^	10	UV-A (25%), UV-B (5%) laser, visible light (70%), 310–600 nm	To correlate clinical symptom scores with possible changes in Langerhans cells of the nasal mucosa induced by ultraviolet irradiation	The irradiation effect was positive, however, no effect was observed on Langerhans cells or other cells of the nasal mucosa immune system.	Open clinical study	High
Cingi et al., 2010^14^	79	UV-A (25%), UV-B (5%) laser, visible light (70%), 310–600 nm	To investigate the effectiveness of phototherapy treatment in patients with AR using TNSS.	Phototherapy can be an effective modality in the treatment of AR, especially when the drugs used are contraindicated and/or show insufficient efficacy	Prospective, randomized, blind, placebo-controlled	Medium
Csoma et al., 2006^4^	13	PUVA laser 305–440 nm	To investigate the efficacy of endonasal PUVA light in the treatment of AR and its effect on skin testing.	Phototherapy plus UV-A is also an effective modality in the treatment of AR.	Open clinical study	Medium
Koreck et al., 2005^2^	49	UV-A (25%), UV-B (5%) laser, visible light (70%), 310–600 nm	To assess whether phototherapy, using a combination of UV-B, UV-A and visible light, is effective in the treatment of AR.	Phototherapy is an effective modality for the treatment of AR and immune-mediated diseases of the mucous membranes.	Randomized, double blind	Low
Csoma et al., 2004^1^	15	XeCL laser UV-B 308 nm	To investigate the clinical efficacy of UV-B irradiation in AR.	It suggests that the XeCL UV-B laser may be a new therapeutic tool in AR.	Open clinical study	High

UV-A, ultraviolet-A; UV-B, ultraviolet-B; AR, allergic rhinitis; TNSS, total nasal symptom scores; PUVA, ultraviolet-A plus; XeCL, xenon chloride.

The assessed symptoms were rhinorrhea (12 studies, 449 participants), sneezing (11 studies, 424 participants), nasal pruritus (10 studies, 409 participants) and nasal obstruction (12 studies, 449 participants).

**Figure 3:**
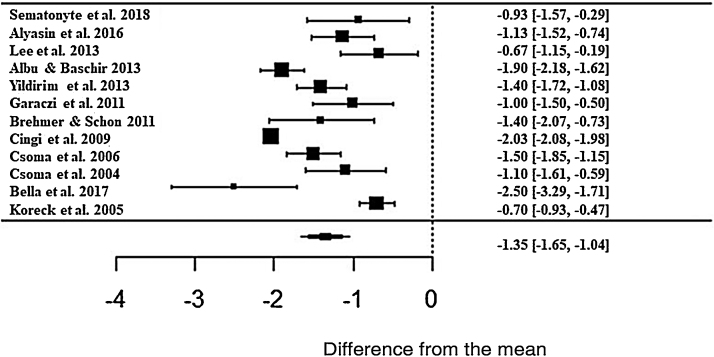
Forest plot of the nasal symptom rhinorrhea evaluated in 12 studies according to the self-reported analog scale. The graph shows the values of the accumulated effect size and for each study with the respective confidence intervals. The size of the squares in the effect bars reflects the weight of the studies.

The meta-analysis result showed a positive effect of phototherapy for the treatment of AR. Patients treated with phototherapy showed 1.35-fold lower mean values ​​in the self-reported analog scale than untreated patients (z = −8.71; SE = 0.1546; *p* < 0.0001; CI = −1.65; −1.04 ) for the rhinorrhea symptom (Fig. 3). Although the effect size is significant, there was a high heterogeneity between studies, with I^2^ = 91.84%. The variance between the studies was true and significant (Q = 224.32, *p* < 0.0001).

**Figure 4:**
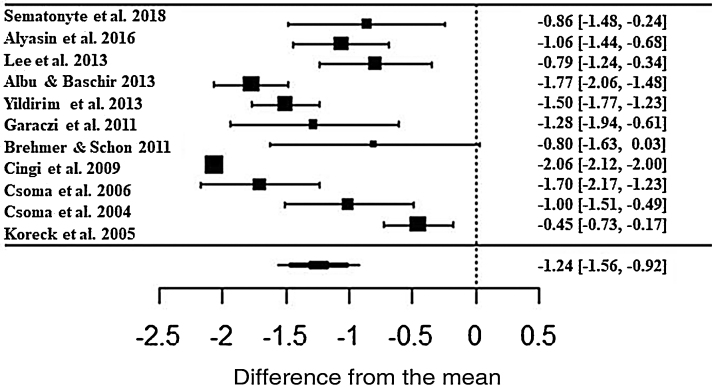
Forest plot of the nasal symptom sneezing evaluated in 11 studies according to the self-reported analog scale. The graph shows the values of the accumulated effect size and for each study with the respective confidence intervals. The size of the squares in the effect bars reflects the weight of the studies.

In the assessment, patients treated with phototherapy in relation to the rhinorrhea symptom showed significant improvements in the scale (ES• = −1.24; z = −7.56; SE = 0.1636; *p* < 0.0001; CI = −1.56; −0.92) (Fig. 4). For this symptom, there was also a high heterogeneity between the studies (I^2^ = 91.43% and Q = 217.4; *p* < 0.0001).

**Figure 5:**
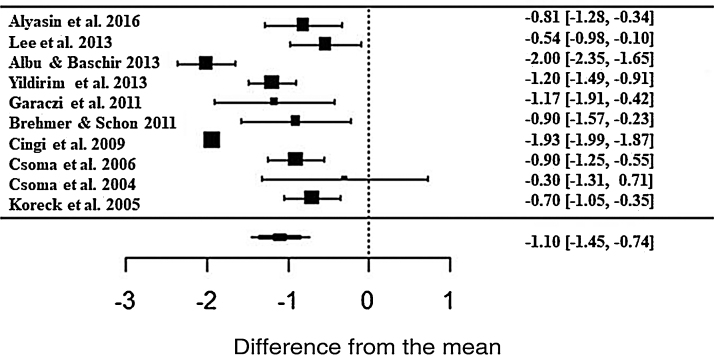
Forest plot of the symptom nasal pruritus evaluated in 10 studies according to the self-reported analog scale. The graph shows the values of the accumulated effect size and for each study with the respective confidence intervals. The size of the squares in the effect bars reflects the weight of the studies.

The phototherapy also showed consistent results and significant improvements for nasal pruritus; however, with ES• = −1.10 (z = −6.03; SE = 0.1821; *p* < 0.0001; CI = −1.45; −0.74) of improvements according to the self-reported scale (Fig. 5), with the heterogeneity at I^2^ = 91.43% and significant variation between the studies (Q = 164.95; *p* < 0.0001).

**Figure 6:**
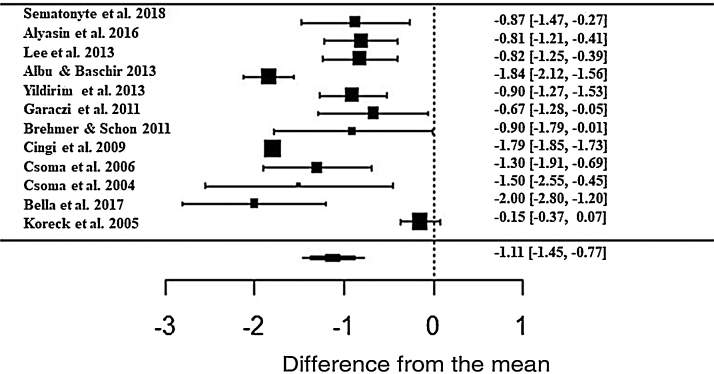
Forest plot of the symptom nasal obstruction evaluated in 12 studies according to the self-reported analog scale. The graph shows the values of the accumulated effect size and for each study with the respective confidence intervals. The size of the squares in the effect bars reflects the weight of the studies.

Regarding the nasal obstruction symptom, patients also showed significant improvement, improving 1.11 in relation to the beginning of treatment (z = -6.40; SE = 0.1736; *p* < 0.0001; CI = −1.45; −0.77) (Fig. 6). As with the other symptoms, there was a high heterogeneity between the studies (I^2^ = 91.88%; Q = 226.52 and *p* < 0.0001).

**Figure 7:**
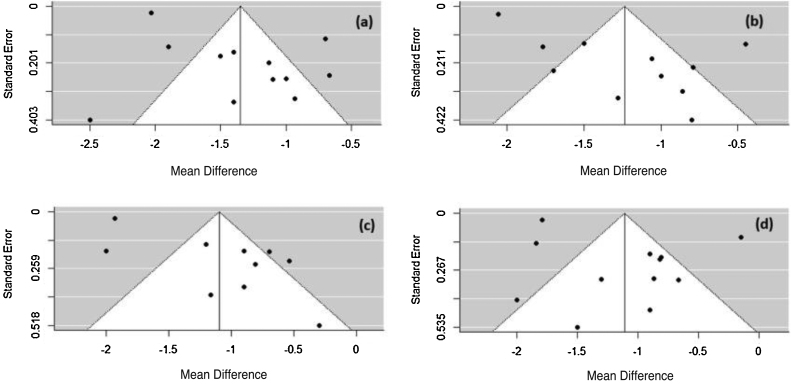
Funnel plot of the analyzed symptoms (a) rhinorrhea, (b) sneezing, (c) nasal pruritus, and (d) nasal obstruction.

The Rosenthal method calculates the mean number of studies that are needed to reduce the significance of the obtained effect size. For all symptoms, N was less than 1, so the meta-analysis is not robust and only one study with N > 5k + 10 (k number of studies included in the meta-analysis) could change the significance of the observed size effect for the considered AR symptoms. To verify the possibility of publication bias, a visual evaluation of the funnel plot was performed. The plot asymmetry indicates publication bias (Fig. 7).

**Figure 8:**
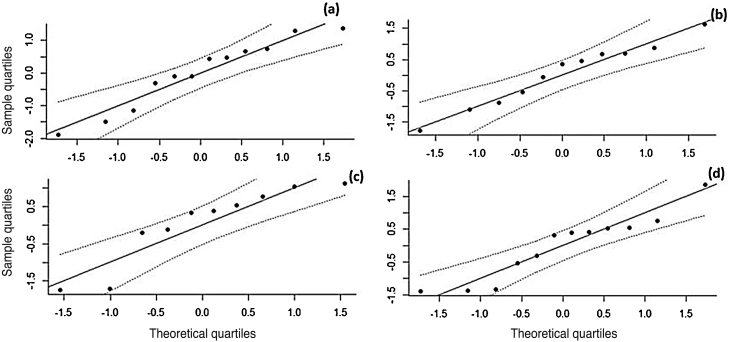
Q–Q plot of the analyzed symptoms (a) rhinorrhea, (b) sneezing, (c) nasal pruritus, and (d) nasal obstruction.

The effect size values ​​follow a normal distribution for the analyzed AR symptoms (Fig. 8). Regarding the analyzed symptoms, there was a high heterogeneity among the studies, as demonstrated by the I^2^ statistic, proposed by Higgins and Thompson (rhinorrhea I^2^ = 91.84%, Sneezing I^2^ = 91.43%, Nasal Pruritus I^2^ = 91.43%, Nasal Obstruction I^2^ = 91.88%). Aiming to explain the observed heterogeneity, subgroup analyses were performed regarding the type of AR, type of study, study pairing and blinding in the studies. The symptoms of perennial AR improved more significantly than seasonal AR when treated with phototherapy for all the considered symptoms (Table 2). The AR type moderator generally explained more than 50% of the variation observed between the studies (R^2^). For all symptoms except rhinorrhea, the randomized studies showed a greater decrease in AR symptoms. The division into randomized and nonrandomized studies explained between 43% and 49% of the variation observed between the studies (Table 2). Paired studies also showed different effect sizes than unpaired ones, with the explanation of variation between the studies reaching 94% in the case of nasal pruritus. Unpaired studies showed greater improvement in AR symptoms, except for nasal pruritus. Non-double-blind studies showed better symptom reduction values, except for nasal pruritus. The explanation values ​​(R^2^) of the variation between the studies for the double blind and non-double blind moderators were between 50% and 55% (Table 2).

**Table 2:** Heterogeneity values (I^2^), percentage of variation explained by the moderates (R^2^) and the effect size for perennial allergic rhinitis (AR) and seasonal allergic rhinitis (AR); study design; pairing and blinding in the studies; in the symptoms of rhinorrhea, sneezing, nasal pruritus and obstruction after the intervention.

	Rhinorrhea	Sneezing	Pruritus	Obstruction
**Type of AR**				
I^2^ group	88.24%	88.99%	89.32%	88.30%
R^2^	59.37%	61.74%	56.88%	61.99%
Q model	**73.8457**	**54.9171**	**34.2273**	**40.33**
Perennial	**−1.4947**	**−1.3587**	**−1.2595**	**−1.2614**
Seasonal	**−1.2474**	**−1.1598**	**−1.0105**	**−0.9955**
**Randomized study**				
I^2^ group	90.70%	90.42%	87.20%	91.81%
R^2^	48.99%	48.89%	45.62%	43.79%
Q model	**76.1853**	**54.0509**	**44.9975**	**38.8202**
Randomized	**−1.1773**	**−1.3399**	**−1.3584**	**−1.1907**
Non-randomized	**−1.5052**	**−1.1374**	**−0.8207**	**−1.0187**
**Paired study**				
I^2^ group	75.90%	83.57%	49.59%	87.32%
R^2^	63.93%	56.65%	94.00%	50.60%
Q model	**158.186**	**80.4247**	**199.863**	**72.8902**
Paired	**−1.0886**	**−1.0598**	**−0.8825**	**−0.9278**
Unpaired	**−1.8567**	**−1.6678**	**−1.8266**	**−1.3521**
**Double blind study**				
I^2^ group	90.69%	90.83%	90.54%	91.25%
R^2^	55.48%	55.28%	50.09%	54.01%
Q model	**71.7681**	**52.0908**	**33.0588**	**37.8885**
Double blind	**−1.2352**	**−1.2846**	**−1.1861**	**−1.0836**
Non-double blind	**−1.4929**	**−1.2002**	**−1.0195**	**−1.1356**

Values with *p* < 0.001 are shown in bold.

The assessed studies were carried out in Europe (Germany: 1 article; Hungary: 5 articles; Lithuania: 1 article; Romania: 1 article), the Middle East (Iran: 1 article; Turkey: 2 articles; Israel: 1 article) and Asia (South Korea: 1 article). It was observed that the geographic region, the climate and the temperature in these places did not interfere with the response to treatment, when evaluated by meta-regression. Regarding the meta-regression, only the rhinorrhea symptom (Table 3) was influenced by the time of treatment (days × weeks = “Time”). Therefore, regarding the rhinorrhea symptom, it was observed that the moderator factor time of treatment obtained significant results, that is, it explained part of the variation between the observed effect sizes. The meta-regression for rhinorrhea considering the moderator factors time, weeks, initial dose and final dose explained 25.89% (R^2^) of the heterogeneity, which went from 91.84% to 86.90%.

**Table 3:** Scores of the meta-regression analysis for the rhinorrhea symptom in patients with allergic rhinitis (AR). Considering the moderators time (days times weeks), weeks (number of weeks), initial dose and final exposure dose.

Parameter	Estimate	SE	z-value	_min_CI	_max_CI	*p*-Value
Intercept	−0.4 141	0.6795	−0.6094	−1.7459	0.9178	0.4554
Time	**0.0313**	**0.0147**	**2.1255**	**0.0024**	**0.0601**	**0.0335**
Weeks	−0.4498	0.2576	1.7459	−0.9547	0.055 1	0.0808
Initial dose	−0.1189	0.6909	−0.1720	−1.4731	1.2353	0.8634
Final dose	−0.0177	0.5395	−0.0328	−1.0751	1.0397	0.9738

Values with *p* <  0.001 are shown in bold.

## Discussion

The therapeutic properties of lasers and their analgesic, anti-inflammatory and healing effects have been studied since their discovery (1960). Their use in dermatology is already well established and the use of phototherapy in the treatment of AR was based on these principles.^2^, ^4^, ^10^ Previous studies have shown that T lymphocyte apoptosis in skin diseases reduces the number and function of dendritic cells and increases immunomodulatory cytokines,^26^ facts observed in other therapies previously established for AR, such as topical glucocorticoids or immunotherapy.^20^

In recent years, non-coherent light sources, such as LED and broadband lamps, have become common in phototherapy treatments. The advantages of LEDs include the safety considerations of the laser, easy use at home, the ability to irradiate a large area of ​​tissue at once, the possibility of wearable devices and a much lower cost per mW.^18^, ^19^

In our analysis, we observed that although the studies had a variety of protocols (light spectrum wavelengths, time of therapy per session, dosimetry in joules, time of treatment per week and number of weeks of intervention), of sample groups and climate, phototherapy showed to be capable of promoting significant improvements in the main symptoms of AR, as demonstrated in the subgroup analysis.

Studies carried out before this meta-analysis investigated the effects of phototherapy as treatment for AR. The first one carried out a literature review and the second performed a literature review with meta-analysis until July of 2014.^5^, ^15^ Due to the absence of a meta-analysis conducted with a robust methodology, our study aimed at better standardization in data reporting, with risk of bias assessment using Cochrane tools and analysis of articles with good methodological designs, although it was not possible to use only randomized studies. Thus, we aimed to gather the best evidence regarding the use of phototherapy for the treatment of AR.

It was observed that the number of studies is small and, therefore, the meta-analysis is still not very robust. However, the effect sizes showed a normal distribution and the results indicated that, despite the significant effect, there was high heterogeneity in the analyzed studies. The considered factors were the use of light spectrums with different frequencies (ranging from ultraviolet to red and infrared), different light sources, time of treatment and specific protocols for each phototherapy device. These factors were assessed by meta-regression and were not considered to be the cause of the heterogeneity observed in the studies.

Other causes can influence heterogeneity, such as clinical factors (patient profile, type of intervention used, definition of the outcome) and methodological factors, such as variation between study designs.^29^ Part of the heterogeneity, regarding the effect size, was explained by the type of rhinitis (perennial or seasonal), type of the study (RCT and non-randomized clinical trials), study pairing (paired and unpaired studies) and blinding (double blind and non-double blind studies). Methodological differences were assessed in the subgroup analysis and were responsible for part of the decrease in heterogeneity and confirmed that phototherapy is effective in AR treatment.

Regarding the analyzed symptoms, the symptomatological efficacy of phototherapy in perennial AR showed a better response when compared to treatment for seasonal AR. In contrast, Cho et al.,^5^ identified in their sample that the effects of phototherapy on AR symptoms were more evident in patients with seasonal AR. As observed in our study, better efficacy in perennial allergic rhinitis seems to make more sense due to the inflammatory alterations this type of rhinitis causes in the nasal turbinates, so that phototherapy can more effectively provide an inflammatory remodeling of the nasal mucosa, as it occurs with prolonged topical corticosteroid use. Therefore, phototherapy might be a good alternative for patients who often require prolonged use of medications to control AR.

As for the treatment protocol, regarding the time of therapy and the monitoring of symptoms post-treatment, flaws were observed, since all studies were completed after the course of therapy, which makes the side effects of long-term regular endonasal treatment unknown. Therefore, the structural and biochemical alterations supposedly induced by the procedure, which would reduce inflammation in the surrounding tissue, could return to the original condition after the treatment with phototherapy, as it occurs after the use of medication and immunotherapy, with the return of symptoms. However, considering the lack of medium and long-term references regarding the effects of endonasal phototherapy, it is not possible to make such assertion. A decrease in nasal mucosa moisture was observed in some studies as an adverse effect during therapy.^8^

The evaluated studies were carried out in Europe, the Middle East and Asia. It was observed that the geographic region, the climate and the temperature in these places did not interfere with response to treatment. However, studies with a larger population sample and at different climates are necessary for better evaluation. It is also necessary to carry out studies with a more adequate methodological design, with a sample and monitoring of the therapy effects in the long term. After this systematic review, it is believed that the lack of robust studies in relation to phototherapy and the existence of so many other drug treatment alternatives for AR have led to a very early discredit regarding this therapeutic option, without further understanding of its advantages and disadvantages. It is also worth reflecting on the possibility of positive results with this therapy, which could go against the interests of large pharmaceutical companies that are always investing in research to prove the effectiveness of their drugs.

The following can be pointed out as limitations of this study: the small number of articles with randomized clinical trials for evaluation and increased risk of bias in most studies. Regarding the quality of the clinical trials, there is a great diversity regarding the methodological designs, few places where research is carried out in this area and the number of samples is small, which makes it difficult to obtain randomized clinical intervention studies with adequate allocation and blinding procedures.

## Conclusion

Considering the effect size and the statistical significance attained in our study, rhinophototherapy showed to be an effective treatment for the reduction of nasal symptom scores triggered by AR.

## Conflicts of interest

The authors declare no conflicts of interest.
